# Biological targeted therapy for lupus nephritis–the role of BLYS and APRIL

**DOI:** 10.1080/0886022X.2025.2561791

**Published:** 2025-11-17

**Authors:** Xin Yang, Siyi Liu, Xiu-Juan Liu, Dan Wen, Yan Zhou, Ying Zeng, Longzhu Li, Qing Deng, Yu Wang, Qinkai Chen, Jinlei Lv

**Affiliations:** a Department of Nephrology, the First Affiliated Hospital of Nanchang University, Nanchang, Institute of molecular immunology for kidney disease of Nanchang University, Nanchang City, Jiangxi, China; b Department of Nephrology, the 908th Hospital of the People’s Liberation Army Joint Logistics Support Force, The Great Wall Affiliated Hospital of Nanchang University, Nanchang City, Jiangxi, China

**Keywords:** B-cell activating factor BAFF, a proliferation-inducing ligand APRIL, telitacicept, lupus nephritis, systemic lupus erythematosus

## Abstract

Lupus nephritis (LN) is a frequent and serious complication of systemic lupus erythemosus (SLE). Both innate and adaptive immunity play essential roles in the initiation and progression of LN. B-lymphocyte stimulator (BLYS) and a proliferation-inducing ligand (APRIL), members of the tumor necrosis factor superfamily, bind to receptors such as B-cell activating factor receptor 3 (BR3), transmembrane activator and CAML interactor protein (TACI), and B-cell maturation antigen (BCMA). BLYS and APRIL are often overexpressed in LN, showing a positive correlation with disease activity. Therapeutic targeting of BLYS/APRIL has demonstrated efficacy in reducing LN activity. Traditional immunosuppressants often have multiple contraindications and side effects due to their broad immune-suppressing effects. Recently, B-cell-targeting agents, including rituximab, belimumab, as well as telitacicept have shown promising efficacy and reduced side effects in LN treatment. Telitacicept, a novel biological agent, has been approved firstly effective for SLE treatment in China. This review focus on the role of BLYS and APRIL in LN development and progression, alongside clinical data on the targeted therapies of above biologicals.

## Graphical Abstract

**Figure:**
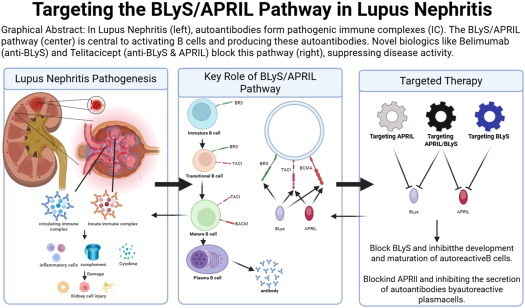

## 1. Introduction

**Figure 1.**
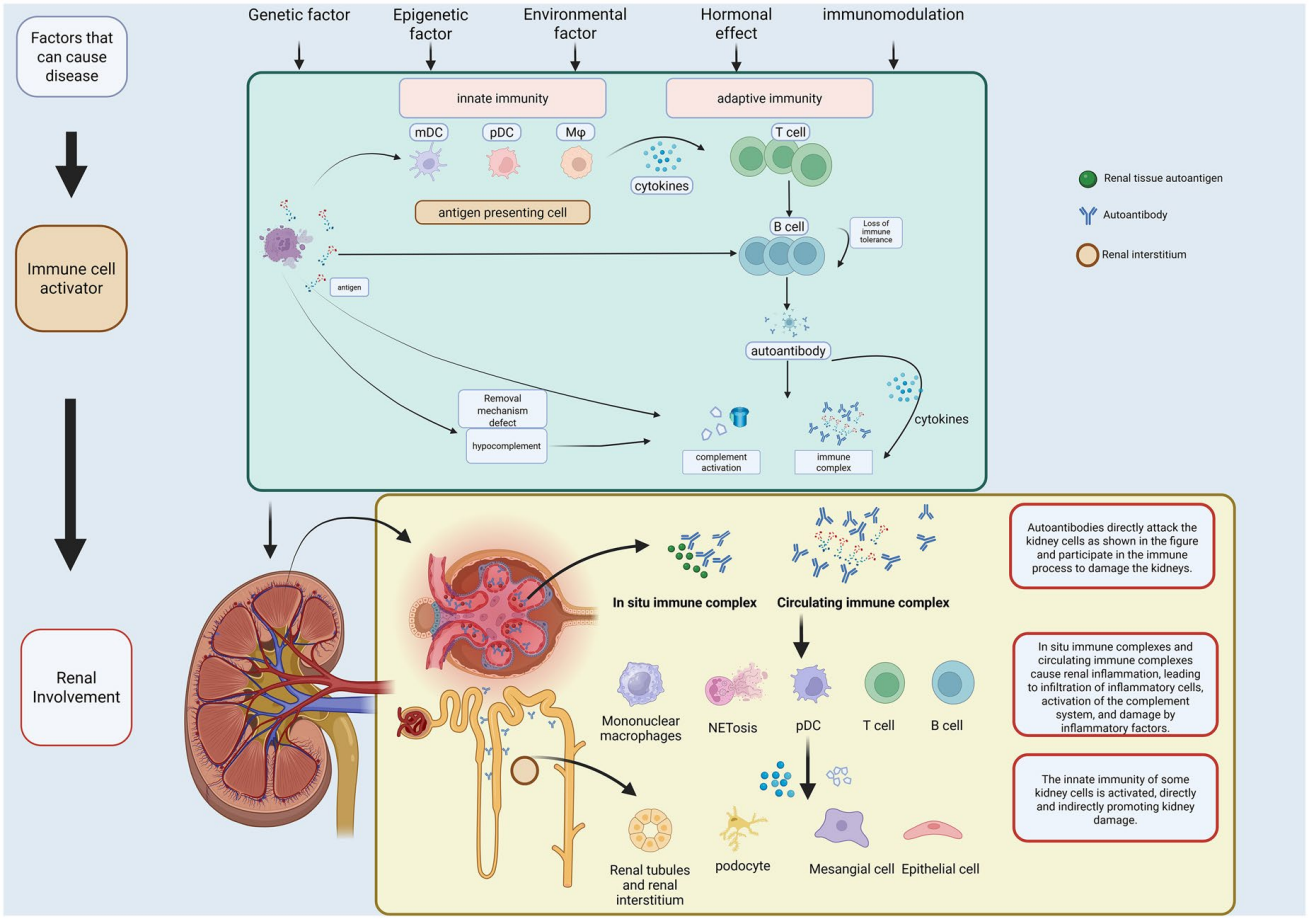
The pathogenesis of LN.

Systemic lupus erythematosus (SLE) is a typical autoimmune disease affecting multiple organ systems. Approximately 35%–60% [1] of SLE patients develop kidney disease, presenting as abnormal urinalysis (hematuria or proteinuria) or renal dysfunction, termed as LN. LN is a frequent and severe complication of SLE which substantially increasing patient mortality. Among patients with lupus nephritis, approximately 5-22% ultimately progress to end-stage kidney disease (ESKD), necessitating dialysis or transplantation [2]. For SLE patients, environmental factors including ultraviolet exposure, dietary habits, gut microbiota changes, sex hormone levels, and genetic predisposition, can disrupt immune balance. This disruption promotes excessive cytokine production, abnormal T and B lymphocyte activation, autoantibody generation, immune complex formation, and subsequent tissue inflammation and organ damage [2]. Circulating and *in situ* immune complexes, formed by B cell derived autoantibodies binding to self-tissues and renal tissues, deposit in glomerular capillaries, furthermore, these deposits activate the complement system, triggering immune responses such as cytokine production, ultimately causing kidney inflammation or LN [3] (Figure 1). LN manifests diverse histological lesions involving glomeruli (e.g., endocapillary hypercellularity, wire loops, crescents), tubulointerstitium (inflammation, atrophy), and vasculature. Recent studies indicate that toll-like receptors (TLR) 7 and 9 [4], along with the NLRP3 (NOD-like receptor thermal protein domain associated protein 3) receptor in innate immunity system, are critical in the pathogenesis and progression of LN [5]. For LN clinical diagnosis, kidney biopsy remains the gold standard, offering guidance for treatment and prognosis based on pathological activity and chronicity. LN is pathologically classified into six types based on kidney biopsy findings. Clinical manifestations vary by type, reflecting pathological differences, and range from mild urinalysis abnormalities to nephrotic syndrome, rapid progressive nephritis, and other conditions.

In clinical practice, LN treatment is guided by renal biopsy results and traditionally involves immunosuppressive agents and immunomodulators which included corticosteroids, cyclophosphamide, mycophenolate mofetil, hydroxychloroquine, and calcineurin inhibitors, such as cyclosporine (CsA) and tacrolimus (TAC) [6]. Studies indicated that cyclophosphamide and mycophenolate mofetil are highly effective for induction and maintenance therapy in type III and type IV LN [6]. However, the side effects and limited efficacy of traditional immunosuppressive agents remain a concern. LN is a major cause of mortality in SLE patients, and outcomes for dialysis and kidney transplants in ESKD remain poor [7]. Timely control of LN can improve the 10-year survival rate from 46% to 95%, according to research [8]. Current LN treatments face challenges including high incidence, safety concerns with immunosuppressive therapies, especially the side effect of corticosteroids and limited efficacy of standard regimens. Thus, preserving kidney function, promptly initiating effective induction and remission therapies, delaying disease progression, lowering morbidity and mortality, minimizing recurrence, and optimizing steroid and immunosuppressive dosage remain critical goals in LN treatment.

**Figure 2.**
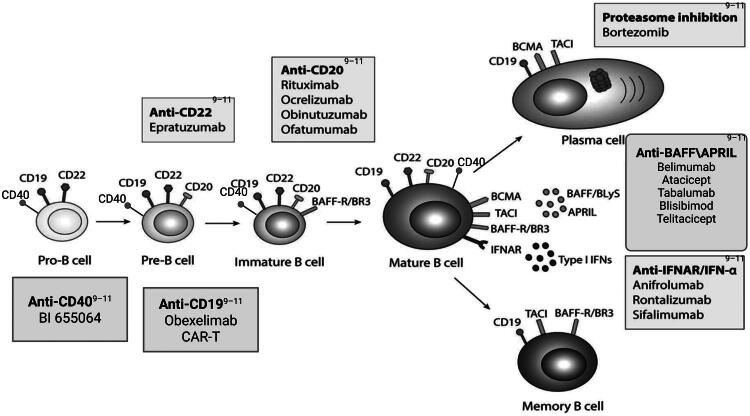
Targeted B-cell therapy-related targets and drugs.

In recent years, targeted B-cell therapies have gained widespread application in autoimmune disease treatment. The example includes monoclonal antibodies targeting CD20 on B-cells (e.g., rituximab, obinutuzumab), biologics targeting B-cell activating factor (e.g., belimumab, telitacicept), and signal transduction inhibitors like Bruton’s tyrosine kinase inhibitors (BTK inhibitors, e.g., tofacitinib) [9] (Figure 2). These therapies offer new options for managing immune-mediated kidney diseases, particularly lupus nephritis.

CD20-targeting therapies include rituximab and obinutuzumab. rituximab is a chimeric antibody with murine and human components, while obinutuzumab is fully humanized. These therapies primarily target and deplete CD20+ on B-cells, thus reducing B-cell derived plasma cells, autoantibody production as well as inflammation [10]. The mechanism lays that Anti-CD20 monoclonal antibodies bind to the CD20 antigen on the surface of B-cells and deplete B-cells through the effect of antibody-dependent cell-mediated cytotoxicity (ADCC), complement-dependent cytotoxicity, additionally direct apoptosis. These effects significantly reduces autoantibody production, immune complex formation, and glomerular damage, thereby improving kidney function and alleviating kidney damage. A multicenter retrospective study showed that rituximab treatment in LN patients significantly improved kidney function and reduced disease activity [11]. The 2023 updated European League Against Rheumatism (EULAR) guidelines recommend rituximab for the treatment of LN patients who do not respond well to standard immunosuppressive therapies. The guidelines emphasize that, in these refractory cases, combining rituximab with other biologics (such as belimumab) may have a synergistic effect [12]. However, the pivotal LUNAR trial [13], which compared rituximab plus background therapy (mycophenolate mofetil and corticosteroids) to placebo plus background therapy in active LN, did not meet its primary efficacy endpoints. This discrepancy between real-world evidence and the randomized controlled trial highlights the ongoing debate regarding the precise role and optimal use of rituximab in LN management, particularly in patients refractory to conventional therapies.

Beyond rituximab, the humanized anti-CD20 monoclonal antibody obinutuzumab has shown promise in LN. The NOBILITY trial [14] demonstrated superior renal response rates compared to placebo in combination with standard therapy. Potential advantages of obinutuzumab over rituximab include its humanized structure (potentially lower immunogenicity), enhanced ADCC, and direct B-cell killing. Further studies are ongoing to define its place in therapy.

Belimumab is a fully human, recombinant IgG2λ monoclonal antibody that targets B lymphocyte stimulator (BLyS). It inhibits BLyS binding to B-cell activating factor receptor (BAFF-R), transmembrane activator and CAML interactor protein (TACI), and B-cell maturation antigen (BCMA), thereby suppressing B lymphocyte differentiation, survival, and antibody secretion. This mechanism effectively controls lupus progression and its renal manifestations. Supported by robust phase III clinical trials, belimumab has been approved for treating SLE. It also demonstrates efficacy in controlling LN progression, reducing relapses, and maintaining an acceptable incidence of adverse events [15–17].

Compared to belimumab, the novel biologic telitacicept targets both APRIL and BlyS, offering a theoretical advantage in treating LN and other autoimmune kidney diseases. Recent clinical trials confirm that telitacicept reduces disease activity in SLE patients, controls kidney disease progression, and exhibits good safety [18]. This finding offers new prospects for LN treatment.

Investigating the roles of BlyS and a proliferation-inducing ligand (APRIL) has gained increasing importance due to the complexity of SLE and LN and the limitations of current treatments, The following section provides a detailed discussion of the roles of APRIL and BLyS in LN pathogenesis, along with recent advances in targeted therapies and related clinical trials. This section aims to elaborate on the key roles of these molecules in LN treatment and explore innovative therapeutic strategies grounded in current scientific insights. The double-targeted biologicals seek to provide new insights and directions for future LN treatment strategies.

## 2. BLYS and APRIL

### 2.1. Normal development of B-cells

**Figure 3.**
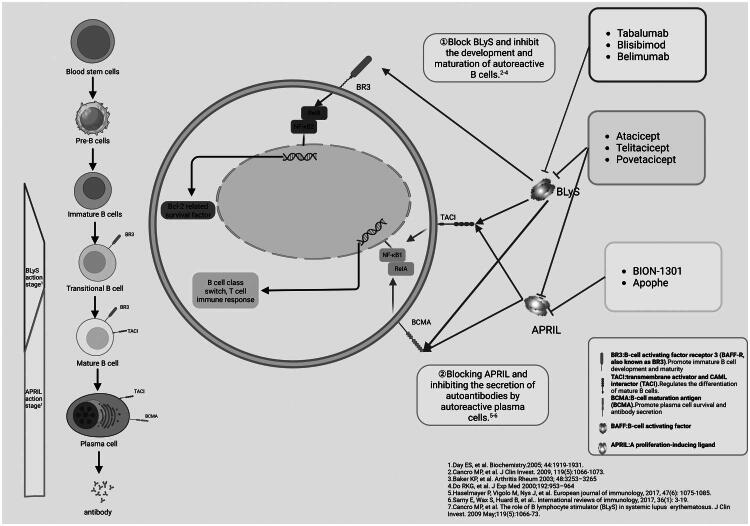
B Cell development and APRIL, BLyS-related signaling pathways. Abbreviations: BLyS, B lymphocyte stimulator; APRIL, a proliferation-inducing ligand; BCMA, B-cell maturation antigen; TACI, transmembrane activator and CAML interactor; BAFF-R, B-cell activating factor receptor.

B-cells develop from hematopoietic stem cells in the bone marrow, progressing through pro-B, pre-B, and immature B-cell stages. Immature B-cells migrate to peripheral immune organs *via* the bloodstream and lymphatic circulation, where they differentiate into marginal zone and follicular B cells. Marginal zone B-cells are critical for early humoral immunity, rapidly producing IgM antibodies against exogenous antigens without requiring T-cell assistance. Follicular B-cells in the germinal centers of the spleen and lymph nodes are activated by T-cells in response to specific antigens. They then differentiate into long-lived memory B-cells and plasma cells. These cells produce high-affinity antibodies and persist in secondary lymphoid organs or the bone marrow [19] (Figure 3).

### 2.2. BLYS and APRIL

BLYS and APRIL play crucial regulatory roles during B-cell development and maturation [20]. Both cytokines belong to the tumor necrosis factor superfamily. They can exist on the cell surface as type II transmembrane proteins or be cleaved by furin protease to release a soluble trimeric form [21–23]. BLyS and APRIL are crucial for B-cell survival, antibody class switching, germinal center maintenance, both T-cell dependent and independent antibody responses, and T-cell co-stimulatory functions [24,25].

BLyS is also expressed by various cell types, including monocytes, macrophages, activated neutrophils, T-cells, and dendritic cells [22,26]. Its expression and secretion are enhanced by inflammatory factors such as interleukine (IL)-2, tumor necrosis factor-alpha, interferon-gamma, and IL-10 [22,24,27],. APRIL is a key immune regulatory molecule produced by various cells, including monocytes, macrophages, dendritic cells, polymorphonuclear cells, neutrophils [23,28], eosinophils [29], megakaryocytes [30], and human intestinal epithelial cells. Furthermore, specific TLR ligands, such as poly I:C and CpG, can induce monocyte-derived dendritic cells to produce APRIL. Additionally, APRIL is also expressed in certain tumor tissues, where it promotes the proliferation of malignant tissues [31].

BLyS and APRIL bind to multiple receptors, including BR3, TACI and BCMA. Specifically, APRIL and BLyS share TACI and BCMA as their receptors, but only BLyS can selectively bind to BR3 [21,32]. The interaction between these ligands and receptors differs in affinity. BLyS has a higher affinity for BR3 than for TACI or BCMA, whereas APRIL shows stronger affinity for TACI and BCMA [33]. Additionally, membrane-bound BLYS can bind to TACI. Notably, the signals and effects mediated by BLYS and APRIL are variable, depending on the receptor type they bind to and the characteristics of the target cells. BR3 is highly expressed on transitional and mature B cells [34]; TACI is present on activated/mature B cells [19]; and BCMA is restricted to plasma cells and memory B cells [35] (Figure 3).

Ligand-receptor binding activates the canonical NF-κB pathway *via* TRAF adaptor proteins, mediating critical B cell function. TACI binds both BLyS and APRIL to activate NF-κB signaling [36]. This receptor regulates B cell activation thresholds, facilitates antibody class switching (notably to IgA), and promotes plasma cell differentiation while preventing excessive proliferation [36]. BCMA exhibits higher affinity for APRIL than BLyS. BCMA sustains long-term plasma cell survival in bone marrow niches, enabling persistent antibody secretion [35]. Its engagement sustains long-lived plasma cell survival through NF-κB activation, thereby maintaining antibody secretion and humoral memory [37,38]. BAFF-R signaling upregulates anti-apoptotic factors (A1, Bcl-xL) [39] to support B cell survival and maturation through transitional stages [34]. In addition, both BR3 and TACI are involved in B-cell antibody class switching [40], and BR3 is also implicated in B-cell selection, survival, and germinal center formation [25]. Dysregulation of either receptor contributes to loss of B cell tolerance in autoimmune settings.

During LN development, immune complexes activate monocytes, macrophages, and plasmacytoid dendritic cells, inducing the production of cytokines, including pro-inflammatory factors TNF and IL-6 [41,42, and key B-cell survival factors BLyS [43]. Elevated BLYS levels promote the survival and expansion of autoreactive B cells in SLE, supporting long-term survival of antibody-secreting plasma cells. This process elevates autoantibody levels, establishing a positive feedback loop that drives disease progression. In a healthy immune system, plasma cells receive survival signals from factors such as APRIL, allowing long-lived plasma cells to persist for decades and generate specific monoclonal antibodies [44], which are critical for vaccine-mediated protection. However, in patients with SLE, long-lived plasma cells persist and secrete pathogenic antibodies, contributing to refractory autoimmune conditions. These long-lived plasma cells exhibit substantial resistance to conventional therapies, including high-dose glucocorticoids, cyclophosphamide, mycophenolate, and rituximab. Studies on SLE treatment highlight that the synergistic effects of BLyS and APRIL are vital for maintaining the survival of autoreactive B cells and long-lived plasma cells, which persistently secrete autoantibodies, forming immune complexes that drive tissue inflammation and organ damage.

## 3. The immune mechanism of LN

In the pathogenesis of LN, apoptotic cells release nucleic acids (DNA and RNA) that are recognized by antigen-presenting cells. These antigen-presenting cells detect and present antigens *via* pattern recognition receptors (PRRs) triggering immune responses [45]. Specifically, pDCs detect nucleic acids using TLRs (TLR7 and TLR9) [46]. TLR7 detects single-stranded RNA, while TLR9 recognizes unmethylated CpG DNA, leading to type I interferon (IFN-α) production [12]. After mDCs engulf apoptotic material, they mature and stimulate T cells to produce IL-17 and activate naïve T cells. SLE patients also exhibit defects in the clearance of apoptotic cells, particularly macrophage dysfunction, leading to the accumulation of apoptotic cells and their components, which results in persistent activation of antigen-presenting cells [47]. In LN, T and B cells lose self-tolerance, resulting in autoimmune responses. Neutrophils also undergo a unique form of cell death, known as NETosis, releasing neutrophil extracellular traps (NETs). This release of nuclear content may generate self-antigens that sustain autoimmune inflammation [48]. These autoantibodies bind self-antigens, forming circulating immune complexes and *in situ* complexes in the kidneys. This activates the complement system, triggering localized inflammation and tissue damage. (Figure 1)

Glomerular injury initiates when immune complexes deposit within capillary walls, activating complement cascades through C1q/C3 binding that directly damage cellular constituents [49]. Endothelial cells undergo activation through membrane attack complex (C5b-9)-mediated lysis and interferon-α-induced expression of adhesion molecules (VCAM-1/ICAM-1), promoting leukocyte recruitment and microthrombosis [50]. Concurrently, mesangial cells proliferate and expand extracellular matrix in response to deposited immune complexes, progressively obliterating capillary lumens [51]. In LN, podocytes sustain direct injury from autoantibodies and participate in immune processes. Damaged podocytes activate innate immunity *via* TLR expression and function as APCs by upregulating MHC class II molecules and costimulatory markers (CD80/CD86), thereby triggering T-cell responses. Furthermore, podocytes collaborate with parietal epithelial cells to promote crescent formation in glomeruli [52] (Figure 1).

Tubulointerstitial pathology evolves through TLR7/9-mediated recognition of nucleosomes within proximal tubules, triggering epithelial-mesenchymal transition characterized by α-smooth muscle actin expression and TGF-β-driven collagen deposition [53,54]. Chemotactic mediators including MCP-1/CCL2 recruit macrophages that establish a self-perpetuating inflammatory circuit through IL-6/TNF-α secretion, inducing tubular atrophy and peritubular capillary rarefaction [55]. Progressive fibrosis is further accelerated by hypoxia-induced HIF-1α activation and protein overload-mediated TLR4/NF-κB signaling, which stimulate connective tissue growth factor production. Tubulointerstitial inflammation severity and tubulointerstitial chronicity are independent predictors of renal failure in lupus nephritis ([HR] 2.2, [95% CI] 1.3–3.6; *p* = 0.002) [56].

### 3.1. BLYS and APRIL play a key role in B cell immunity in the occurrence and progression of LN

The pathogenesis of LN involves several pathogenic processes related to B cell abnormalities. Abnormalities in both central and peripheral tolerance mechanisms lead to the generation of autoreactive B cells, which, under abnormal T-B cell interactions, exhibit prolonged survival, enhanced somatic hypermutation, and class switch recombination, thereby increasing the pathogenicity, differentiation, and survival of plasma cells. This process is regulated by the upregulation of key B cell factors, including BLYS, IL-6, and IL-21. The upregulation of these factors promotes B cell activation and antibody production, thereby exacerbating the autoimmune response and disease progression. In the circulating and infiltrating B cell subsets, B cell abnormalities are characterized by an increase in peripheral plasma cells and memory B cells, along with a decrease in naive B cells. Memory B cells exhibit reduced expression of FcγRIIb, lowering the reactivation threshold and making them more easily activated upon encountering antigens or inflammatory signals. Although the proliferation rate of these memory B cells is lower, they are resistant to conventional immunosuppressive drugs, making them more prone to reactivation during disease relapse, which leads to the production of large amounts of autoantibodies and exacerbates disease symptoms. These abnormal mechanisms collectively contribute to the onset and progression of LN [57–59].

In this process, BLYS and APRIL play key roles. These are critical regulators of B cell survival and development; Studies in BLYS transgenic mouse models have identified autoimmune manifestations resembling SLE, further confirming that BLYS overexpression plays a role in promoting the development of such diseases [60].

Early studies have demonstrated that plasma levels of APRIL and BLYS are significantly higher in SLE patients compared to healthy individuals [61–63]. Salazar-Camarena et al. reported that serum levels of BLYS and APRIL were elevated in SLE patients and correlated with disease activity indices, including Mex-SLEDAI. They further observed an increased proportion of memory B cells and plasma cells in the peripheral blood of SLE patients [64]. Petri et al. conducted a prospective evaluation of 245 SLE patients, revealing that circulating BLYS levels correlated with anti-double-stranded DNA antibody titers and SELENA-SLEDAI scores (another measure of SLE activity), further supporting the association between BLYS levels and SLE disease activity [65]. Becker-Merok et al. observed that SLE patients with elevated BLYS levels exhibited higher SLEDAI scores and increased C-reactive protein levels [66]. Hegazy et al. compared SLE patients, RA patients, and healthy controls and found that APRIL levels were significantly higher in SLE patients, showing a positive correlation with disease severity [67].

Treamtrakanpon et al. reported that proteinuria in LN patients is associated with elevated APRIL levels, and serum APRIL levels correlate with resistance to immunosuppressive therapy [68]. Moreover, a study detected significantly elevated levels of APRIL and BLYS in the glomeruli of LN patients. It observed that CD68-positive cells in the glomeruli upregulate APRIL expression, whereas interstitial inflammatory cells also produce BLYS and APRIL [69].

## 4. Targeted therapies against APRIL and BLYS show favorable efficacy in LN

### 4.1. Targeting BLYS therapy

In therapeutic research on SLE, biologics targeting BLYS have shown promising potential. Tabalumab, a human IgG4 monoclonal antibody, neutralizes both membrane-bound and soluble forms of BLYS. However, in two phase III clinical trials for SLE, tabalumab did not meet the primary efficacy endpoints. Although its safety profile was comparable, with most adverse events being mild to moderate, higher rates of depression and suicidal ideation were observed in the tabalumab group. Consequently, in 2014, the developer announced the discontinuation of tabalumab as a therapeutic option for SLE [70,71]. Therefore, the sponsor officially abandoned the development of tabalumab as a treatment for SLE in 2014.

Another BLYS inhibitor, Blisibimod, did not achieve the primary efficacy endpoints in phase II and III clinical trials for SLE. However, it showed partial efficacy in reducing glucocorticoid use, restoring complement levels, and lowering proteinuria in patients [72,73]. Its safety profile was favorable, with common adverse events including upper respiratory tract infections, urinary tract infections, injection site erythema, and diarrhea.

In comparison, belimumab, another BLYS-targeting biologic, significantly improved clinical symptoms, serological markers, and renal function indicators in active SLE compared to the placebo group in two phase III clinical trials, BLISS-52 and BLISS-76, with good tolerability [15,16]. Tabalumab and blisibimod failed to meet primary endpoints in Phase 3 SLE trials. Potential reasons for this divergence in outcomes compared to belimumab include differences in: (1) Target Neutralization: Belimumab primarily binds soluble BLyS, while tabalumab targeted both soluble and membrane-bound forms. The biological significance of membrane-bound BLyS neutralization and potential off-target effects remain unclear. (2) Trial Design/Population: Differences in patient populations, background therapies, and endpoint definitions may have contributed. (3) Pharmacodynamics: Variations in drug structure, binding affinity, half-life, and depth of BLyS inhibition could influence efficacy. The success of belimumab underscores the importance of soluble BLyS blockade in SLE/LN, while the failures highlight the complexity of target engagement and trial execution.”

Supported by high-quality clinical trials and extensive clinical practice, the 2021 KDIGO guidelines recommended belimumab as an adjunct to standard treatment regimens [74]. By 2024, the KDIGO guidelines further highlighted the role of belimumab in both induction and maintenance therapy for LN, recommending it for patients with renal relapse or high risk of renal failure, with a suggested treatment duration exceeding 2.5 years [75]. These findings suggest that BLYS-targeted therapy, particularly Belimumab, demonstrates promising efficacy and safety in treating SLE and LN, making it an effective therapeutic option. These findings underscore the critical role of the BLyS pathway in SLE and its complication LN, emphasizing the therapeutic potential of targeting this pathway (Table 1).

**Table 1.** Clinical trials of biologic therapies targeting BLyS in lupus nephritis.

Investigational Drugs	Types of Research	Research Subjects	Grouping Information	Dosage and Administration	Observation Endpoint	Treatment Efficacy	Adverse Events	Serious Adverse Events	Common Adverse Reactions	Citation
Tabalumab	52-week Phase III multicenter, randomized, double-blind, placebo-controlled study	SLE patients; meet ≥4/11 ACR criteria, ANA positive, SELENA-SLEDAI ≥6 (*n* = 1124)		Subcutaneous injection, initial loading dose of 240 mg at week 0, followed by 120 mg every 2 weeks (Q2W) or every 4 weeks (Q4W).	The proportion of patients achieving SLE Responder Index 5 (SRI-5) [1] at week 52.	120 Q2W: 38.4% of patients achieved SRI-5 (relative to placebo 27.7%, *p* = 0.002); 120 Q4W: 34.8% of patients achieved SRI-5 (relative to placebo 27.7%, *p* = 0.051).	The overall adverse event rate was close to 82%, similar to the placebo group.	Serious adverse events accounted for approximately 12%-19%, similar to the placebo group.	Injection site reactions, depression, and suicidal thoughts were more common.	Merrill JT et al. Ann Rheum Dis 2016;75:332–340
	52-week multicenter, randomized, double-blind, placebo-controlled study.	SLE patients; meet ≥4/11 ACR criteria, ANA positive, SELENA-SLEDAI ≥6 (*n* = 1164).	Tabalumab 120 mg Q2W(*n* = 372) Tabalumab 120 mg Q4W(*n* = 376) , Placebo control group (*n* = 376)	Subcutaneous injection, with a loading dose of 240 mg at week 0, followed by 120 mg every 2 weeks (Q2W) or every 4 weeks (Q4W).	The proportion of patients achieving SLE Responder Index 5 (SRI-5) at week 52.	The SRI-5 response rate for 120 Q2W was 31.8%, and for 120 Q4W was 35.2%, with no significant difference compared to placebo.	The incidence of treatment-emergent adverse events (TEAEs) was 81.1-82.3%.	Death: 0.5-0.8%, severe infections: 4.9-5.2%	Death: 0.5-0.8%, severe infections: 4.9-5.2%	Isenberg et al. 2016
Blisibimod	52-week clinical phase III double-blind, randomized controlled study	SLE patients, positive for antinuclear antibodies (ANA) or anti-double-stranded DNA antibodies, with SELENA-SLEDAI ≥ 10 (*n* = 442)	Blisibimod 200 mg weekly (QW) (*n* = 245). Placebo control group (*n* = 197)	Subcutaneous injection, administered once a week, 200 mg per dose	Proportion of patients reaching the SLE Responder Index-6 (SRI-6) [1] at week 52	The primary endpoint of SRI-6 did not show a significant difference, but significant effects were observed in reducing corticosteroid use, proteinuria, and biomarker response.	Upper respiratory infections, urinary tract infections, injection site erythema/reaction, and diarrhea were the most common. The overall adverse event rate was 69.8% (Blisibimod) vs 64.8% (placebo).	The incidence of serious adverse events was 13.1% (Blisibimod) vs 17.3% (placebo).	Erythema at the injection site, injection site reactions, upper respiratory tract infections	Merrill et al. (2018). Annals of the Rheumatic Diseases, doi:10.1136/annrheumdis-2018-213032
	Phase IIb randomized, placebo-controlled, dose-range study	Adult SLE patients, positive for anti-double-stranded DNA or antinuclear antibodies, SELENA-SLEDAI score ≥6 (*n* = 547)	Blisibimod 200 mg QW. Placebo-controlled group	Subcutaneous injection, once a week, 200 mg per dose	Achieved SLE Responder Index-5 (SRI-5) at week 24	No significant increase in SRI-5 response rates was observed with any dose of Blisibimod compared to the placebo group.In patients receiving the highest dose of Blisibimod, the SRI^1^ response rate was higher than the placebo group from week 16 to week 24, reaching statistical significance at week 20 (*p* = 0.02).	No significant imbalance was observed in terms of serious adverse events, infections, deaths, or malignancies.	No statistical difference was observed in the occurrence of serious adverse events between the two groups.	Upper respiratory tract infections, urinary tract infections.Injection site reactions, such as erythema and reactions (including pain, swelling, etc.), diarrhea	Furie RA, et al. (2015). Annals of the Rheumatic Diseases, 74(9), 1667–1675. doi:10.1136/annrheumdis-2013-205144. PMID: 24748629
Belimumab	Phase III, double-blind, randomized controlled trial (BLISS-76)	Adult SLE patients who are ANA-positive or anti-dsDNA-positive with a SELENA-SLEDAI score >6 (*n* = 819)	Placebo group (*n* = 275) 1 mg/kg Belimumab (*n* = 271) 10 mg/kg Belimumab (*n* = 273)	Intravenous injection, at days 0, 14, and 28, then every 28 days thereafter.	SRI response rate at 52 weeks^1^	Compared to the placebo group, the SRI response rate in the 10 mg/kg dose group was significantly higher (43.2% vs 33.5%, *p* = 0.017); the SRI response rate in the 1 mg/kg dose group was also higher than in the placebo group, but the difference was not statistically significant.	The incidence of adverse events was similar to that of the placebo group.	Serious adverse events, including infections, laboratory abnormalities, malignancies, and death, were comparable across groups.	Upper respiratory infections, headache, urinary tract infections, joint pain	Furie et al. 2011
	Phase III double-blind randomized controlled trial (BLISS-52)	Adult SLE patients with positive ANA or anti-dsDNA, SELENA-SLEDAI score >6 (*n* = 867)	Belimumab 1 mg/kg (*n* = 289). Belimumab 10 mg/kg, (*n* = 290). Placebo group (*n* = 288)	Intravenous injection on days 0, 14, 28, and then every 28 days until week 48	SRI response rate at 52 weeks^1^	At week 52, the SRI response rates were significantly higher in the Belimumab 1 mg/kg group (51% of patients) and 10 mg/kg group (58% of patients) compared to the placebo group (44% of patients).	Similar between the Belimumab group and the placebo group	8% of patients in the 1 mg/kg group reported serious infections, 4% in the 10 mg/kg group, and 6% in the placebo group. - No malignant diseases reported.	Nausea, diarrhea, fever, headache, fatigue, and infusion reactions.	Navarra et al. 2011
	104-week phase III multicenter, randomized, double-blind, placebo-controlled trial	Adult (≥18 years) patients with active lupus nephritis, confirmed by renal biopsy within the past 6 months with active lupus nephritis (Class III or IV). Evidence of at least one instance of immunosuppressive treatment due to SLE disease activity in the past 2 years, and baseline urine protein/creatinine ratio (UPCR) ≥1.0. (*n* = 448)	Belimumab 10 mg/kg group (*n* = 224), placebo group (*n* = 224)	Intravenous administration, given on days 0, 14, 28, and then every 28 days until week 100.	Primary endpoint: Renal response at week 104^2^. Secondary endpoints: Complete renal response^3^ at week 104, time to renal-related events or deat^3^.	At week 104, more patients in the Belimumab group achieved a primary renal response^2^ (43% vs 32%, *p* = 0.03) and a complete renal response (30% vs 20%, *p* = 0.02) compared to the placebo group. Patients receiving Belimumab had a lower risk of renal-related events or death compared to those receiving placebo (hazard ratio 0.51, *p* = 0.001).	The Belimumab group and the placebo group were similar.	Similar between the Belimumab group and the placebo group	Upper respiratory tract infections, urinary tract infections, shingles, bronchitis, nasopharyngitis, headaches, nausea, rashes, etc.	Furie R, Rovin BH, Houssiau F, et al. Two-Year, Randomized, Controlled Trial of Belimumab in Lupus Nephritis. N Engl J Med. 2020;383(12):1117–1128. Doi:10.1056/NEJMoa2001180.

Annotations.

1.The SLE Responder Index (SRI) response rate at week 52, defined as a reduction of ≥4 points in SELENA-SLEDAI score, no new BILAG A organ domain scores, and no worsening in Physician’s Global Assessment (PGA) score.

2.Primary renal response (major efficacy response for renal outcomes), including urine protein/creatinine ratio ≤0.7, estimated glomerular filtration rate (eGFR) not lower than 80% of pre-flare value or ≥60 mL/min/1.73 m², and no use of rescue therapy.

3. Complete renal response (urine protein/creatinine ratio <0.5, eGFR not lower than 90% of pre-flare value or ≥90 mL/min/1.73 m², and no use of rescue therapy).

### 4.2. Targeting APRIL therapy

Targeting APRIL has shown therapeutic potential in LN studies. BION-1301, also known as Zigakibart, is a novel humanized monoclonal antibody targeting APRIL. It is currently in clinical trials, primarily for IgA nephropathy (IgAN), and has not yet received market approval [76]. Although BION-1301 primarily targets IgA nephropathy, it may also have applications in other APRIL-associated diseases, such as LN . In foundational LN research, Huard et al. used a murine APRIL IgG1 antibody, Apophe, to block interactions between soluble APRIL and its receptors BCMA and TACI. Experimental results showed that Apophe-treated mice had better outcomes than controls, including reduced IgM and IgG autoantibody production, significantly lower proteinuria at six months, reduced mortality, decreased IgM deposition, less PAS-positive material, and smaller glomerular cell volumes [77]. Additionally, APRIL-deficient lupus model mice showed lower incidences of nephritis and reduced autoantibody production [78]. These animal studies highlighted the potential of APRIL blockade to alleviate LN-related pathology and provided key evidence for developing APRIL-targeted therapies. The positive outcomes in these animal models underscore the need for further exploration of such therapies to provide more effective treatment options for SLE and LN patients.

### 4.3. Targeting APRIL and BLYS

Targeted therapy against BLYS and APRIL has proven effective in treating LN. Several drugs, including atacicept, telitacicept, and povetacicept, have demonstrated potential in clinical trials. Atacicept, a fusion protein, consists of the extracellular ligand-binding domain of TACI and the Fc portion of human IgG, designed to block APRIL and BLYS interactions with their receptors. A double-blind, placebo-controlled study evaluated the efficacy and safety of atacicept in preventing flares in moderate to severe SLE patients, with patients randomly assigned to receive 75 mg atacicept, 150 mg atacicept, or a placebo. Although the atacicept 150 mg group study was terminated early due to two patient deaths, it showed positive effects compared to placebo in reducing flare rates (OR: 0.48, *p* = 0.002) and prolonging time to first flare (HR: 0.56, *p* = 0.009). Most adverse events were mild to moderate [79]. In the phase IIb multicenter, randomized controlled trial ADDRESS II, atacicept showed good efficacy compared to placebo, particularly in subgroups with high baseline disease activity and serologically active disease, where efficacy was more significant [18]. Atacicept use increased the probability of achieving low disease activity status in lupus compared to the control group [80]. The clinical efficacy and safety of atacicept for treating SLE need further verification through more clinical trials.

Povetacicept (ALPN-303) is a novel dual antagonist of APRIL and BLYS with enhanced activity. In cell-based studies, povetacicept more effectively inhibited APRIL and BLYS activity compared to traditional TACI-Fc fusion proteins and specific APRIL and BLYS inhibitors, effectively blocking B cell proliferation, differentiation, and immunoglobulin (Ig) secretion. In mouse models of immune diseases, compared to traditional treatments such as anti-CD20 monoclonal antibodies and conventional dual-target inhibitors (WT TACI-Fc), povetacicept more effectively reduced serum immunoglobulin titers and the number of antibody-producing cells. In the mouse LN model, povetacicept significantly improved survival rates, reduced proteinuria and anti-double-stranded DNA antibody titers, improved blood urea nitrogen levels, and effectively inhibited glomerulonephritis and renal immunoglobulin deposition. In non-human primate studies, povetacicept showed good tolerance, high serum exposure, and significant reduction in IgM, IgA, and IgG levels [81]. Povetacicept is currently undergoing Phase I clinical trials for SLE, including studies on SLE, glomerulonephritis, and cytopenias, with successful completion of Phase I trials for SLE. Based on preliminary pharmacodynamic data from the RUBY-1 study, the every-four-week dosing regimen of povetacicept showed stronger activity and greater convenience than traditional BAFF/APRIL inhibitors. These findings suggest that povetacicept may become an effective new treatment option, particularly for autoimmune diseases such as SLE and LN [82].

Telitacicept is a novel fusion protein developed by Rongchang Pharmaceuticals, composed of the extracellular soluble domain of the TACI and the crystallizable fragment (Fc) of human immunoglobulin G [83].The TACI domain in the telitacicept molecule binds to and neutralizes two B cell activation molecules, BlyS and APRIL, effectively inhibiting overactive B cells and plasma cells in autoimmune diseases, thus limiting their production of autoantibodies [83]. Compared to belimumab, which targets only BlyS, the dual-target mechanism of telitacicept theoretically offers a broader inhibitory effect, potentially performing better in controlling the progression of autoimmune diseases. Telitacicept is administered *via* subcutaneous injection, with the recommended dose being 160 mg per injection. Clinical studies indicate that adverse reactions to telitacicept are generally mild, including upper respiratory tract infections, urinary tract infections, and injection site reactions, with overall good safety and tolerability.

Telitacicept has demonstrated promising therapeutic effects in clinical studies for SLE and LN. In a Phase I, randomized, single-blind, placebo-controlled study involving 12 SLE patients, the experimental group received 180 mg of telitacicept subcutaneously once weekly for 4 weeks, with continuous observation for 84 days. After treatment, the experimental group showed overall disease stability compared to the placebo group, with a decrease in B cell count and immunoglobulin levels, as well as an increase in complement C3 and C4 levels. Regarding safety, most adverse events in the experimental group were mild to moderate; however, the incidence of lumbar rib pain, shingles, and rashes was slightly higher in this group [84]. A subsequent Phase IIb, multicenter, randomized, double-blind, placebo-controlled clinical trial involving 249 patients with active SLE demonstrated that different doses of telitacicept (80 mg, 160 mg, 240 mg) significantly improved the SLE response index 4 response rate at 48 weeks compared to placebo (Table 2). A decrease in the SELENA-SLEDAI score further confirmed the positive impact of telitacicept on SLE. Regarding safety, the incidence of adverse events and serious adverse events was similar between the experimental and control groups, with common adverse events including upper respiratory tract infections and injection site reactions [85]. The Phase III study results presented in the ACR Convergence 2022 abstract further support the safety and efficacy of telitacicept in SLE treatment, showing that the response index 4 response rate in the telitacicept group was significantly higher than the placebo group from week 4 and continued through week 52^86^.

**Table 2.** Clinical trials of biologic therapies targeting BlyS and APRIL in lupus nephritis.

Investigational Drugs	Types of Research	Research Subjects	Grouping Information	Dosage and Administration	Observation Endpoint	Treatment Efficacy	Adverse Events	Serious Adverse Events	Common Adverse Reactions	Citation
Atacicept	Phase II/III 52-week randomized, double-blind, placebo-controlled, parallel-group multicenter trial	Adult patients with moderate-to-severe systemic lupus erythematosus (SLE) with active disease and positive anti-double-stranded DNA antibodies (*n* = 306)	Placebo group (*n* = 103). Atacicept 75 mg group: (*n* = 102). Atacicept 150 mg group: (*n* = 101).	Subcutaneous injection. During the first 4 weeks, patients received two injections per week (a total of 8 injections), followed by once-weekly injections until week 52.	Primary efficacy endpoints: flare rate and time to first SLE flare. Secondary efficacy endpoints: overall BILAG improvement, quality of life assessments, and changes in laboratory parameters. Safety endpoints: incidence of adverse events and changes in laboratory safety parameters.	The Atacicept 75 mg group showed no significant differences compared to placebo in reducing SLE flare rates or delaying time to first flare. In contrast, the Atacicept 150 mg group significantly reduced SLE flare rates (*p* = 0.002) and prolonged time to first SLE flare (*p* = 0.009), with notable improvements in laboratory parameters associated with SLE disease activity.	Adverse events included upper respiratory tract infections, urinary tract infections, injection site reactions, nausea, and headaches, with comparable incidence rates.	Two cases of death occurred, both in patients receiving atacicept 150 mg treatment.	Upper respiratory tract infections, urinary tract infections, injection site reactions, nausea, and headaches.	Isenberg D, Gordon C, Licu D, et al. Efficacy and safety of atacicept for prevention of flares in patients with moderate-to-severe systemic lupus erythematosus (SLE): 52-week data (APRIL-SLE randomized trial). Ann Rheum Dis. 2015;74:2006–2015. doi:10.1136/annrheumdis-2013-205067
	A 24-week randomized, double-blind, placebo-controlled, multicenter phase IIb clinical study.	Patients with active autoantibody-positive systemic lupus erythematosus (SLE), SLEDAI-2K score ≥6, meeting at least four criteria of the revised ACR classification for SLE, positive for antinuclear antibodies (Hep-2 ANA ≥1:80) and/or anti-double-stranded DNA antibodies (≥30 IU/mL), with a disease duration of at least 6 months.	Placebo (*n* = 100), Atacicept 75 mg (*n* = 102), Atacicept 150 mg (*n* = 104)	Once a week, subcutaneous injection for 24 weeks	Primary endpoint: Improvement in SLE Responder Index (SRI)-4 at week 24.	SRI-4 response rate: Atacicept 75 mg was 57.8% (compared to placebo group OR: 1.78, *p* = 0.045), 150 mg was 53.8% (OR 1.56, *p* = 0.121), placebo was 44.0%.	Comparable to the control group	Comparable to the control group	Injection site reactions、Upper respiratory tract infections	Isenberg DA, Petri M, Kalunian K, et al. Efficacy and safety of subcutaneous tabalumab in patients with systemic lupus erythematosus: results from ILLUMINATE-1, a 52-week, phase III, multicentre, randomised, double-blind, placebo-controlled study. Ann Rheum Dis. 2016;75(2):323-331. doi:10.1136/annrheumdis-2015-207653
	24-week Phase 2b, multicenter, randomized, double-blind, placebo-controlled study	Patients with high disease activity (HDA) at baseline (SLEDAI-2K ≥ 10) (*n* = 158)	Placebo group (*n* = 52), Atacicept 75 mg group (*n* = 55), Atacicept 150 mg group (*n* = 51)	Once a week, subcutaneous injection for 24 weeks	Primary: SLE Responder Index (SRI)-4 and SRI-6 responses.Treatment goal (T2T) endpoints: Low disease activity (LDA, SLEDAI-2K ≤ 2), low lupus disease activity state (LLDAS), and remission (clinical SLEDAI-2K = 0, prednisone ≤5mg/day, physician’s global assessment <0.5).	At week 24, compared to placebo, atacicept 150 mg significantly increased the achievement of LDA (OR 3.82, *p* = 0.007) and LLDAS (OR 5.03, *p* = 0.018). Remission increase was observed but did not reach statistical significance (OR 3.98, *p* = 0.095).	–	–	Injection site erythema, injection site reactions, upper respiratory tract infections	Morand EF, et al. “Rheumatology.” 2020;59:2930–2938. doi:10.1093/rheumatology/keaa029
Telitacicept	52-week randomized, double-blind, placebo-controlled phase III clinical trial. 48-week clinical phase 2b trial.	Among those aged 18 to 65, patients with positive ANA and/or anti-dsDNA and a SELENA-SLEDAI score of 8 or higher (*n* = 335) had SLE.	SLE patients aged between 18 and 65 years, with positive ANA and/or anti-dsDNA, and a SELENA-SLEDAI score ≥ 8. (*n* = 335)	Telitacicept 160 mg or placebo, subcutaneously administered once weekly, in combination with standard treatment, for 52 weeks.	The primary endpoint was the SLE response index 4 (SRI4) response rate at week 52.	The Telitacicept group achieved an SRI4 response rate of 82.6% at week 52, significantly higher than the 38.1% in the placebo group (*p* < 0.001).	The Telitacicept group was 91.6%, and the placebo group was 84.5%.	The Telitacicept group had 7.2% (12 patients with 17 SAEs), and the placebo group had 14.3% (24 patients with 36 SAEs).	Upper respiratory tract infections, decreased serum IgG, decreased serum IgM, injection site reactions, and urinary tract infections.	Di Wu, Jing Li, Dong Xu, et al. “Telitacicept, a Human Recombinant Fusion Protein Targeting B Lymphocyte Stimulator (BlyS) and a Proliferation-Inducing Ligand (APRIL), in Systemic Lupus Erythematosus (SLE): Results of a Phase 3 Study”. ARTHRITIS & RHEUMATOLOGY. 2022, 74: 4546–4548.
		SLE patients, aged 18 to 65 years, with positive ANA and/or anti-dsDNA, and a SELENA-SLEDAI score ≥ 8 (*n* = 249).	Telitacicept 80 mg (*n* = 62), 160 mg (*n* = 63), 240 mg (*n* = 62), placebo (*n* = 62).	Once weekly, with doses of 80 mg, 160 mg, 240 mg, or placebo, in combination with standard treatment, administered subcutaneously for 48 weeks.	SLE Responder Index 4 (SRI4) response rate at week 48	SRI4 response rate: Telitacicept 80 mg (71.0%, *p* < 0.0001), 160 mg (68.3%, *p* = 0.0001), 240 mg (75.8%, *p* < 0.0001) compared to placebo (33.9%). SELENA-SLEDAI score reduction ≥4 points: Telitacicept 80 mg (75.8%, *p* = 0.003), 160 mg (77.8%, *p* = 0.001), 240 mg (79.0%, *p* < 0.001) compared to placebo (50.0%).	Telitacicept 240 mg (93.5%), 160 mg (92.1%), 80 mg (90.3%), placebo (82.3%). The incidence of adverse events was similar between groups (*p* > 0.05).	Telitacicept 240 mg (12.9%), 160 mg (15.9%), 80 mg (12.9%), placebo (16.1%). The incidence of serious adverse events was similar between groups (*p* > 0.05).	Upper respiratory tract infections, injection site reactions.	ClinicalTrials.gov, Identifier NCT02885610.
	4-week randomized, single-blind, placebo-controlled phase I clinical study.	12 Chinese patients with mild SLE (*n* = 12), 2 males and 10 females.	RCT-18 (180 mg) group: 9 participants, placebo group: 3 participants.	RCT-18 administered at a dose of 180 mg, subcutaneously once a week for a total of 4 doses.	The primary endpoint was the SLEDAI score.	RCT-18 can reduce the levels of immunoglobulin M (IgM) and IgA in serum.	The incidence of moderate and severe infections was higher in the treatment group than in the placebo group, with a total of 89 events.	Three serious adverse events (SAEs) were reported by three patients receiving RCT-18 treatment.	Infection and rash.	Zhao Q, Chen X, Hou Y, et al. Pharmacokinetics, pharmacodynamics, safety, and clinical activity of multiple doses of RCT-18 in Chinese patients with systemic lupus erythematosus. J Clin Pharmacol. 2016;56(8):948–959. doi:10.1002/jcph.686

## 5. Discussion

The evidence synthesized in this review firmly establishes BLyS and APRIL as central mediators in the immunopathogenesis of LN, validating their therapeutic targeting as a rational strategy. Elevated serum and intrarenal levels of these cytokines correlate significantly with histological activity and clinical severity, reflecting their fundamental role in sustaining autoreactive B-cell clones through interactions with TACI, BCMA, and BR3. This signaling axis drives defective B-cell tolerance, enhances survival of autoreactive transitional B cells, and critically maintains long-lived plasma cells within protective bone marrow niches. These plasma cells demonstrate remarkable resistance to conventional immunosuppression and persistently secrete pathogenic autoantibodies, establishing a self-perpetuating cycle of immune complex deposition, complement activation, and renal inflammation.

Therapeutic inhibition of this pathway represents a paradigm shift from broad immunosuppression toward precision medicine. Belimumab, a monoclonal antibody neutralizing soluble BLyS, has demonstrated significant efficacy in reducing renal flares and proteinuria across phase III trials and real-world studies, leading to its inclusion in the KDIGO 2024 recommendations for both induction and maintenance therapy in high-risk LN. However, its exclusive targeting of soluble BLyS leaves membrane-bound BLyS and APRIL signaling intact, potentially explaining incomplete responses in some patients where residual plasma cell activity and APRIL-mediated immunoglobulin production persist.

In this context, dual BLyS/APRIL inhibitors offer a mechanistically advantageous approach. Telitacicept, a TACI-Fc fusion protein, demonstrates broader immunomodulation by simultaneously neutralizing both cytokines. Clinical data from Chinese SLE cohorts reveal significant improvements in SRI-4 response rates and reductions in SELENA-SLEDAI scores compared to placebo, alongside a favorable safety profile characterized primarily by mild-to-moderate upper respiratory infections. Preclinically, its dual blockade achieves superior suppression of immunoglobulin-secreting cells compared to selective BLyS inhibition, potentially offering enhanced control over APRIL-driven IgA/IgG production and plasma cell longevity – key factors in LN pathogenesis.

Emerging agents further refine this dual-target strategy. Povetacicept, an engineered Fc fusion protein with enhanced affinity, demonstrates profound reductions in autoantibodies and glomerular immunoglobulin deposition in murine LN models, outperforming traditional TACI-Fc construct. Its extended half-life supports monthly dosing, improving practicality over weekly regimens. While APRIL-specific inhibitors like BION-1301 show promise in IgA nephropathy, their potential in APRIL-dominant LN subsets remains untested clinically.

Several critical questions must guide future research. First, significant heterogeneity exists in treatment response. Identifying predictive biomarkers—such as high baseline BLyS/APRIL levels, specific autoantibody profiles (e.g. anti-dsDNA, anti-C1q), or renal gene expression signatures—is essential for patient stratification. Second, despite targeting BCMA, deeply resident long-lived plasma cells may evade current biologics. Combinatorial approaches incorporating proteasome inhibitors (e.g. bortezomib) or anti-CD38 antibodies warrant investigation to eradicate this resilient reservoir. Third, optimizing combination strategies is crucial, particularly for refractory disease. The EULAR recommendation of rituximab in non-responders suggests potential synergy between B-cell depletion and BLyS/APRIL blockade; clinical trials evaluating belimumab or telitacicept combined with anti-CD20 therapies are needed. Finally, long-term safety monitoring is imperative, particularly regarding APRIL’s roles in vascular immune inflammation and intestinal immunity.

## Supplementary Material

Abbreviations.docx

## Clinical trial registration

As this study did not involve any clinical trials, there is no trial registration number.
