# Supplementary material for: Biological targeted therapy for lupus nephritis–the role of BLYS and APRIL
Source: Ren Fail. 2025 Nov 17;47(1):2561791. doi: 10.1080/0886022X.2025.2561791 (PMC12624907; doi:10.1080/0886022X.2025.2561791)
Supplement: Abbreviations.docx [file IRNF_A_2561791_SM2828.docx]

**ABBREVIATIONS**

| **Abbreviation** | **Full name** |
| --- | --- |
| ADCC | antibody-dependent cell-mediated cytotoxicity |
| AID | Activation-Induced Cytidine Deaminase |
| ANAs | antinuclear antibodies |
| APCs | antigen-presenting cells |
| APRIL | a proliferation-inducing ligand |
| BCMA | B-cell maturation antigen |
| BIM | B-cell lymphoma-2 interacting mediator of cell death |
| BLIMP-1 | B-lymphocyte-induced maturation protein-1 |
| BLISS-52 | Study of Belimumab in Subjects with SLE 52 |
| BLISS-76 | Study of Belimumab in Subjects with SLE 76 |
| BLYS | B-lymphocyte stimulator, also known as BAFF |
| BR3 | B-cell activating factor receptor 3 (BAFF-R, also known as BR3) |
| BTK | Bruton's tyrosine kinase |
| CD | cluster of differentiation |
| CDC | complement-dependent cytotoxicity |
| CDS | cytosolic DNA sensors |
| CsA | cyclosporin a |
| CSR | class switch recombination |
| CTX | cyclophosphamide |
| CVID | common variable immune deficiency |
| CXCL | C-X-C Motif Chemokine Ligand |
| ESRD | end-stage renal disease |
| EULAR | The European Alliance of Associations for Rheumatology |
| FOB | follicular B-cells |
| ICOS | Inducible T-cell Costimulator |
| IFN | interferon |
| IgG4-RD | IgG4-related diseases |
| IKK | IκB kinase |
| IL | interleukin |
| ITP | Immune Thrombocytopenia |
| KDIGO | **Kidney Disease: Improving Global Outcomes** |
| LN | Lupus nephritis |
| mDCs | myeloid dendritic cells |
| Mex-SLEDAI | Mexican Systemic Lupus Erythematosus Disease Activity Index |
| MMF | mycophenolate mofetil |
| MS | multiple sclerosis |
| MyD88 | Myeloid differentiation factor88 |
| MZB | marginal zone B-cells |
| MΦ | macrophages |
| NETs | neutrophil extracellular traps |
| NLRP3 | NOD-like receptor thermal protein domain associated protein 3 |
| NLRs | NOD-like receptors |
| PAMP | pathogen-associated molecular patterns |
| pDCs | plasmacytoid dendritic cells |
| PRRs | pattern recognition receptors |
| RA | Rheumatoid Arthritis |
| RLRs | RIG-like receptors |
| SHM | somatic hypermutation |
| SLE | Systemic Lupus Erythematosus |
| SRI4 | SLE response index (4) |
| SS | Sjögren's Syndrome |
| TAC | tacrolimus |
| TACI | transmembrane activator and CAML interactor protein |
| Th1/Th17 | Helper T cell |
| THC | Toll/Interleukin-1 receptor homology domain |
| TLR | Toll-like receptor |
| TNF | tumor necrosis factor |
| TRAF | Tumor Necrosis Factor Receptor-Associated Factor |
| XBP-1 | X-box binding protein 1 |
